# A Comparison of the Quality of Meat from Female and Male Californian and Flemish Giant Gray Rabbits

**DOI:** 10.3390/ani10122216

**Published:** 2020-11-26

**Authors:** Tomasz Daszkiewicz, Andrzej Gugołek

**Affiliations:** 1 Department of Commodity Science and Processing of Animal Raw Materials, University of Warmia and Mazury in Olsztyn, Oczapowskiego 5, 10-719 Olsztyn, Poland; 2 Department of Fur-Bearing Animal Breeding and Game Management, University of Warmia and Mazury in Olsztyn, Oczapowskiego 5, 10-719 Olsztyn, Poland; gugolek@uwm.edu.pl

**Keywords:** rabbits, breed, sex, meat quality

## Abstract

**Simple Summary:**

The countries with the highest annual levels of meat consumption consume around 90 kg of meat per person. However, the strong market position of meat is accompanied by changes in consumer preferences resulting from the growing levels of nutritional awareness and nutrition education, as well as greater availability of information about food quality and the link between nutrition and health. As a result, there is a high demand for meat products that have high nutritional value, are original and safe for consumers, and deliver pleasant sensory experiences. According to the literature, rabbit meat is one of such products. Information about the factors that influence the production of high-quality meat is important for producers who want to meet consumer expectations. The aim of this study was to determine the quality of meat from different carcass cuts in female and male rabbits of medium-sized (Californian—CAL) and large (Flemish Giant Gray—FG) breeds. It was found that due to its desirable chemical composition and sensory properties, rabbit meat may be an alternative product on the meat market, but its quality can be affected by the genotype of animals and the anatomical origin of muscles.

**Abstract:**

This study aimed to compare the quality of meat (*Longissimus thoracis et lumborum*—LTL, hind legs—HL) from female and male Californian (CAL) and Flemish Giant Gray (FG) rabbits. The animals were kept outdoor, in a roofed pavilion, in wire mesh cages with a slatted floor and were fed ad libitum a complete pelleted diet. All rabbits were slaughtered at 91 days of age. The meat of CAL rabbits had a higher content of dry matter (*p* < 0.001), protein (*p* < 0.001) and unsaturated fatty acids (*p* = 0.002), higher pH (*p* < 0.001), and higher taste desirability (*p* = 0.021) and tenderness (*p* = 0.046). CAL rabbit meat had also a lower (*p* < 0.001) water to protein (W/P) ratio, lower (*p* < 0.001) shear force values, and lower (*p* = 0.042) taste intensity. The meat of FG rabbits was characterized by lower (*p* < 0.001) water-holding capacity (WHC). The LTL muscle had a higher content of dry matter (*p* = 0.003) and protein (*p* < 0.001), higher L* (lightness) (*p* = 0.011), b* (yellowness) (*p* < 0.001), and C* (chroma) (*p* = 0.010) values, and lower (*p* = 0.015) WHC. Hind leg muscles had higher (*p* < 0.001) fat content, a higher (*p* < 0.001) W/P ratio, and pH (*p* < 0.001). Sex had no effect (*p* > 0.05) on the analyzed meat quality attributes, excluding vitamin A content which was higher (*p* = 0.041) in females. In conclusion, the meat of CAL rabbits slaughtered at 91 days of age can be more mature than the meat of FG rabbits slaughtered at the same age. Furthermore, quality of meat from rabbits of both breeds depends on the anatomical origin of muscles. Further research is needed to identify the possible reasons for the higher vitamin A content of meat from female rabbits which did not differ from the meat of males in terms of other characteristics.

## 1. Introduction

Global rabbit meat production remains relatively low. According to the FAO [1], 980,785,000 rabbits were slaughtered in 2016 around the world, and 1,428,085 tons of rabbit meat were produced (compared with the global meat production of 329,890,425 tons). Asia is the largest rabbit meat producer in the world (approx. 73% of the global market) [2], followed by Europe, Africa, and the Americas whose share of the global market is approximately 20%, 6%, and 1%, respectively. Rabbit meat production has increased in recent years, but not in all regions of the world. The highest increase was noted in Asia, accompanied by a limited increase in Africa and a decrease in Europe, North America, and South America [3]. Differences in rabbit farming are also observed within geographic regions. In Europe, rabbit farming is concentrated in Spain, France, and Italy, representing around 80% of the total EU production [4]. The scale of rabbit farming and the distribution channels of rabbit meat also vary. In the EU, 180 million rabbits are raised for meat, of which around 66% are kept in commercial farms whereas around 34% are reared and consumed in backyard farms or sold directly on local markets [4].

Rabbit meat produced in both intensive and extensive farming systems is valued for its unique properties and high nutritional value, it is recommended by dietitians and is found attractive by many consumers. An analysis of the chemical composition of rabbit meat has revealed that it has a high content of protein (approx. 80% of the energy value) with a high share of essential amino acids (lysine, sulfur-containing amino acids, threonine, valine, isoleucine, leucine, and phenylalanine) compared with most types of meat consumed today [5]. The high biological value of rabbit meat is also determined by its fatty acid composition, with a predominance (approx. 60%) of unsaturated fatty acids (UFAs), and a 27–33% proportion of polyunsaturated fatty acids (PUFAs) [6]. Rabbit meat has a high content of B vitamins, in particular B3 and B12 (100 g of rabbit meat covers 77% and 300% of the daily requirement for vitamins B3 and B12, respectively) [7,8], and a low content of sodium [9], purines and uric acid [8]. According to some researchers [7], rabbit meat can be used in the production of functional foods due to its health benefits.

Large-scale commercial rabbit meat production is based on synthetic lines of rabbits [10] created by crossing breeds and lines characterized by high fertility, high fecundity, and a fast growth rate [11]. Since rabbits weighing from 2 to more than 2.6 kg are in high demand, synthetic lines are represented mostly by rabbits of medium-sized breeds, slaughtered at 9–13 weeks of age [6]. Pure breeds (both large and medium-sized) used for creating synthetic lines are less suitable for intensive farming. However, they can be used in organic farming, which is gaining popularity [12]. An analysis of the genetic potential of the above breeds for producing high-quality meat is important for producers (regardless of production scale or system) who want to meet consumer expectations.

In view of the above, the aim of this study was to compare the quality of the *Longissimus thoracis et lumborum* (LTL) muscle and hind leg (HL) muscles obtained from female and male Californian (CAL) and Flemish Giant Gray (FG) rabbits, slaughtered at 91 days of age.

## 2. Materials and Methods

### 2.1. Materials

The experiment was approved by the local Ethics Committee for Animal Experimentation (Olsztyn, Poland, decision No. 24/2014). The experimental materials comprised 20 CAL rabbits and 20 FG rabbits (10 males and 10 females in each group) from a breeding farm in northern Poland. Animals weaned at 42 days of age were selected for the study. At weaning, the average body weight of CAL and FG rabbits was 986 ± 62 g and 1133 ± 97 g, respectively.

On the farm, the rabbits were kept outdoors, in a roofed pavilion, in wire mesh cages (0.8 × 0.7 × 1 m) with a slatted floor (two animals of the same sex per cage), equipped with automatic feeders and drinkers. The animals were fed ad libitum a complete pelleted diet (Table 1) whose nutritional value corresponded to the requirements of growing meat-type rabbits [13].

The rabbits were slaughtered (according to the standard guidelines for euthanizing rabbits) at 91 days of age, after 24 h fasting. Skinned and eviscerated carcasses were placed in a cold storage chamber at a temperature of 3 °C. After 24 h, chilled carcasses were divided into the following parts: head (cut between the occiput and the atlas), fore part (cut between the 7th and 8th thoracic vertebrae), intermediate part (cut between the 6th and 7th lumbar vertebrae), and hind part (carcass section that remains after the intermediate part had been cut off the fore part). After carcass dressing, the hind legs were removed from the hind part. HL muscles and the LTL muscle from the intermediate part were removed from each carcass, and were subjected to laboratory analyses, which were performed 28–30 h post mortem.

### 2.2. Methods

#### 2.2.1. Chemical Analyses

The content of dry matter (drying at 105 °C to constant weight), total protein (Kjeldahl method, Kjeltec™ 8400 Auto Distillation Unit, FOSS Analytical, Hilleroed, Denmark), crude fat (Soxhlet extraction with diethyl ether as the solvent, Soxtec™ 2050 Auto Fat Extraction System, FOSS Analytical, Hilleroed, Denmark), and minerals as crude ash (incineration at 550 °C to constant weight) in meat samples was determined by standard AOAC methods [14]. The energy value of meat was determined based on protein and fat content, using energy factors for protein and fat of 16.78 and 37.62 kJ/g, respectively [15].

The cholesterol content of the LTL muscle was determined by colorimetric analysis (Epoll-20^®^ spectrophotometer, Poll Ltd. Sp. z o. o., Warsaw, Poland; wavelength of 520 nm), during an enzymatic reaction catalyzed by cholesterol peroxidase [16], using the Pointe Scientific assay kit with the reference standard of 200 mg/100 cm^3^ (Pointe Scientific, Warsaw, Poland).

The content of retinol (vitamin A) and α-tocopherol (vitamin E) in the LTL muscle was determined as described by Hőgberg et al. [17] and Xu [18]. The levels of vitamins were determined by reversed-phase high-performance liquid chromatography (SHIMADZU, Japan; HPLC system), on a Nucleosil C18 column, with methanol/water (95:5, *v:v*) as the mobile phase, a UV detector (326 nm for retinol), and a fluorescence detector (excitation −295 nm, emission −330 nm for tocopherol), flow rate: 1 cm^3^/min., loop: 20 μL.

#### 2.2.2. Meat pH

The pH values of minced samples collected from the LTL muscle and HL muscles were measured (approx. 29 h post mortem) in muscle homogenates (meat to redistilled water ratio of 1:1, *m/v*) with the use of a combination Polilyte Lab electrode (Hamilton Bonaduz AG, Bonaduz, Switzerland) and the 340i pH-meter with a TFK 325 temperature sensor (WTW Wissenschaftlich-Technische Werkstätten, Weilheim, Germany) [15].

#### 2.2.3. Meat Color

The color parameters (L*—lightness, a*—redness, b*—yellowness, C*—chroma) of minced samples collected from the LTL muscle and HL muscles were determined in the CIELAB color space [19]. The values of L*, a*, and b* were measured with the HunterLab MiniScan XE Plus spectrocolorimeter (Hunter Associates Laboratory, Reston, VN, USA) as described by Daszkiewicz and Janiszewski [15]. The final result was the arithmetic mean of three measurements at different points over the surface area of minced meat samples. The value of C* was calculated from the formula: C* = (a*^2^ + b*^2^)^1/2^.

#### 2.2.4. Water-Holding Capacity

Water-holding capacity (WHC) was determined by the filter paper press method [20]. A sample of minced meat (300 mg) was placed on filter paper (Whatman^®^ No 1) between two cover glasses under a pressure of 5 kg and held there for 5 min. The difference between the area of the pressed meat and the wet area on filter paper (determined with a planimeter and expressed in cm^2^) was a measure of exudative juice (WHC).

#### 2.2.5. Fatty Acid Profile

The analysis was performed on samples of intramuscular fat (IMF) extracted by the Soxhlet method from the LTL muscle. Fatty acid methyl esters were extracted by the modified method of Peisker [21] with chloroform:methanol:sulfuric acid (100:100:1 *v*/*v*). Samples of extracted IMF were transferred into glass ampoules with the addition of 1.5 mL of methylated mixture, and the ampoules were sealed in the flame of a burner to melt glass. Next, the ampoules were placed in a thermostat with a temperature of 105 °C for 2 h. Fatty acids were separated by gas chromatography on the VARIAN CP-3800 gas chromatograph (Varian Inc., Palo Alto, CA, USA) equipped with a flame ionization detector (FID) and a capillary column (length—50 m, inner diameter—0.25 mm, liquid phase—CP-Sil 88, film thickness—0.25 μm) [15]. The carrier gas was helium (flow rate—1.2 mL/min). The results are expressed as percentages of saturated and unsaturated fatty acids in the total fatty acid pool in intramuscular fat.

#### 2.2.6. Shear Force

Shear force was measured in LTL muscle samples prepared as described by Honikel [22], using the INSTRON 5542 universal testing machine (Instron, Canton, MA, USA) fitted with a Warner-Bratzler head (500 N, speed 100 mm/min). The final result was the arithmetic mean of measurements of the maximum shear force required to cut four cylinder-shaped meat samples (diameter—1.27 cm, height—2 cm) across the grain [15].

#### 2.2.7. Sensory Analysis

The sensory quality of cooked LTL muscle samples (0.6% aqueous solution of NaCl, 96 °C, 1 h) was evaluated by five panelists who had been trained in the sensory properties of cooked rabbit meat prior to the analysis. A five-point scale (5 points—most desirable, 1 point—least desirable) described by Daszkiewicz et al. [23] was used. The panelists assessed meat cubes (1 × 1 × 1 cm) cut out from the center of each cooked sample. All sensory properties (aroma, taste, juiciness, tenderness) of encoded samples were evaluated during one session. Redistilled water was made available to the panelists for mouth cleansing between samples [15].

### 2.3. Statistical Analysis

Data were analyzed using Statistica software ver. 13.3 (TIBCO Software Inc., Palo Alto, CA, USA). A three-way ANOVA was used to analyze the effects of breed (CAL, FG), sex (female, male), and muscle type (LTL, HL) on the chemical composition and physicochemical properties of meat. Two-way ANOVA was performed for a model where breed and sex were fixed effects exerted on the content of cholesterol, vitamins and fatty acids, and the sensory properties of meat. When interactions between the main factors were not detected (*p* > 0.05), the significance of differences between means in groups was determined by Tukey’s test. When interactions were detected (*p* ≤ 0.05), one-way ANOVA was performed, and group means were compared by Tukey’s test. The significance of differences between means in groups was determined at a significance level of *p* ≤ 0.05.

## 3. Results

### 3.1. Proximate Chemical Composition

An analysis of the effect of breed on the chemical composition of meat (Table 2) revealed that the meat of CAL rabbits had higher (*p* < 0.001) protein content (by 1.2 percentage points) than the meat of FG rabbits. Moreover, the fat content of meat from CAL rabbits was approximately one-fifth higher, compared with FG rabbits, but the difference between group means was not statistically significant (*p* = 0.124). As a result, the meat of CAL rabbits had higher (*p* ≤ 0.001) total dry matter content and a lower (*p* < 0.001) W/P ratio. The higher protein and fat content of meat from CAL rabbits led to its higher (*p* < 0.001) energy value.

Sex had no effect (*p* ≥ 0.389) on the major chemical components content in rabbit meat (Table 2). However, the vitamin A content of meat was nearly two-fold higher (*p* = 0.041) in females than in males (Table 2).

The chemical composition of the analyzed muscles varied depending on their anatomical location (Table 2). HL muscles had higher (*p* < 0.001) fat content. The LTL muscle had higher (*p* < 0.001) protein content (by 1.54 percentage points) and, in consequence, higher (*p* = 0.003) dry matter content and a lower (*p* < 0.001) W/P ratio. The five-fold higher fat content of HL muscles contributed to their higher (*p* = 0.033) energy value.

ANOVA revealed an interaction (*p* = 0.030) between rabbit breed and sex for the fat content of meat, which was approximately two-fold higher (0.99 vs. 0.55%, *p* = 0.049) in male CAL rabbits than in male FG rabbits.

### 3.2. Fatty Acid Profile

The proportion of saturated fatty acids (SFAs) in rabbit meat is presented in Table 3. The effect of breed was noted in the concentrations of capric acid (C10:0) and lauric acid (C12:0), which were higher (*p* = 0.001 and *p* = 0.019, respectively) in the meat of CAL rabbits, and in the concentrations of pentadecanoic acid (C15:0) (*p* < 0.001), margaric acid (C17:0) (*p* < 0.001), stearic acid (C18:0) (*p* = 0.002), and arachidic acid (C20:0) (*p* = 0.010), which were higher in the meat of FG rabbits. Due to the considerable difference in the proportion of C18:0 fatty acid, the meat of FG rabbits had higher (*p* = 0.002) total SFA content.

The effect of sex on the SFA content of meat was noted only in the proportion of C10:0 fatty acid, which was higher (*p* = 0.020) in males (Table 3).

Unsaturated fatty acids (UFAs) profile of rabbit meat is presented in Table 4. The meat of FG rabbits had higher concentrations of myristoleic acid (C14:1) (*p* = 0.024) and arachidonic acid (C20:4) (*p* = 0.019). The meat of CAL rabbits had a higher proportion of palmitoleic acid (C16:1) (*p* = 0.001), total content of monounsaturated fatty acids (MUFAs) (*p* = 0.027), and UFAs (*p* = 0.002). As a result, the meat of CAL rabbits was characterized by higher (*p* = 0.002) MUFA/SFA and UFA/SFA ratios (Table 5).

The UFA profile of meat was similar in males and females, and a significant (*p* < 0.001) difference was noted only in the proportion of gadoleic acid (C20:1) which was higher (by 0.05 percentage points) in males. The significant difference between group means resulted from low variation in the content of this acid (Table 4).

### 3.3. Physicochemical Properties

A comparison of breed types revealed that the meat of CAL rabbits had higher (*p* < 0.001) pH and WHC values (Table 6). The average values of the analyzed physicochemical properties of rabbit meat were similar (*p* ≥ 0.334) in males and females (Table 6). Considerable differences in the physicochemical properties of meat were found between the LTL muscle and HL muscles (Table 6). The LTL muscle was characterized by lower (*p* < 0.001) pH, higher L* (*p* = 0.011), b* (*p* < 0.001), and C* (*p* = 0.010) values, and lower (*p* = 0.015) WHC. An interaction between sex and muscle type (*p* = 0.015) was noted for the value of b*, which was higher in males in the LTL muscle (16.05) compared with HL muscles in males (14.68, *p* < 0.001) and in females (15.01, *p* = 0.004).

### 3.4. Sensory Properties

A sensory analysis revealed that the LTL muscle of CAL rabbits, compared with FG rabbits, was characterized by lower (*p* = 0.042) taste intensity, higher (*p* = 0.021) taste desirability, and higher (*p* = 0.046) tenderness, as confirmed by lower (*p* < 0.001) shear force values (Table 7).

Sex had no effect (*p* ≥ 0.357) on the evaluated sensory attributes of the LTL muscle (Table 7). An interaction (*p* = 0.012) was found between rabbit sex and breed for the taste desirability of meat. The meat of male FG rabbits scored higher (*p* = 0.012) for this attribute than the meat of male CAL rabbits.

## 4. Discussion

### 4.1. Proximate Chemical Composition

The quality of rabbit meat is affected by breeding strategies [24] related to selection for rapid growth [25]. As a result, rabbits reach their slaughter weight within a shorter time, but the quality of their carcasses and meat may vary widely, even when the animals are slaughtered at the same age. In the present study, differences in the quality of meat from CAL and FG rabbits slaughtered at 91 days of age could be expected, since medium-sized and large rabbit breeds differ in growth rate and maturity at slaughter [24]. It was found that the meat of CAL rabbits had lower water content (by 1.49 percentage points) and higher protein content (by 1.2 percentage points). A lower W/P ratio is more desirable because it contributes to higher product yield during meat processing (lower loss of own water, greater ability to hold added water). The meat of CAL rabbits tended to have higher IMF content (by 0.18 percentage points), which also confirms that it was more mature at slaughter. However, it should be noted that IMF content is not the best indicator of meat maturity in rabbits, contrary to other animal species [26], because rabbit meat is very lean [27].

The quality (including proximate chemical composition) of meat from FG rabbits remains insufficiently investigated. Frunza et al. [28] analyzed the quality of samples collected from the *Longissimus dorsi* (LD), *Triceps brachii* (TB), and *Semimembranosus* (SM) muscles of rabbits slaughtered at 11 months of age. They found that the LD muscle from males had the highest protein content (21.70%), the TB muscle of females had the highest proportion of lipids (2.65%), whereas ash content in the analyzed three muscle groups was relatively close, with higher average values in the TB muscle (1.231% in males). In the present study, meat from FG rabbits had higher average protein content and lower fat content. The differences between the present findings and the results obtained by Frunza et al. [28] could be due to considerable differences in the slaughter age of FG rabbits (3 months vs. 11 months). Gondret et al. [29] reported that in rabbit, IMF content increases with age, provided that the age differences are not too small. In turn, fat content is inversely related to the protein and water content of meat [30].

Studies investigating CAL rabbits are more numerous, but they often report incomplete information on the muscle type or composition of meat samples, which consist of different muscles or carcass cuts. Belichovska et al. [31] found that genotype had no significant effect on the proximate chemical composition of HL muscles in CAL and New Zealand White (NZW) rabbits or the crosses between these two breeds. The analyzed meat contained 21.59–22.01% of protein, 2.62–2.88% of fat, and 1.12–1.37 of ash. In a study by Maj et al. [32], the protein content of the *Longissimus* muscle in CAL rabbits slaughtered at 2.5 kg BW (12–13 weeks of age) reached 22.15%, and it was comparable with that noted in NZW rabbits and in (NZW × CAL) × CAL and (CAL × NZW) × NZW crosses. The fat content of meat ranged from 1.60% to 1.70% in CAL and NZW rabbits and (CAL × NZW) × NZW crosses, and it was lower than in (NZW × CAL) × CAL crosses (2.17%) where the CAL genotype predominated. Piórkowska [33] analyzed meat quality in six breeds (NZW, Termond White, Danish White, CAL, Alaskan and Grand Chinchilla) of rabbits slaughtered at 91 days of age and found no interbreed differences in the protein content of meat (*Longissimus* and HL muscles), which ranged from 21.7% in CAL rabbits to 22.4% in Grand Chinchillas. The average fat content of meat ranged from 2.8% to 4.4%, and it was highest in CAL rabbits. The genetic potential for IMF deposition in rabbit breeds and lines is difficult to determine because it has not been thoroughly examined to date, but as demonstrated by Martínez-Alvaro et al. [34], IMF content can be modified by the selection of animals.

In the present study, sex had no significant effect on the proximate chemical composition of rabbit meat, which corroborates previous research [28,35,36]. Our findings suggest that there is an interaction between rabbit breed and sex for IMF content. Similar results were reported by Ortiz Hernández and Rubio Lozano [37] who found that the meat of female CAL rabbits had higher IMF content compared with males, whereas the opposite was observed in NZW rabbits (no differences in the proximate composition of meat were found between male and female Rex and Chinchilla rabbits).

In the current experiment, rabbit muscles had a high content of total protein and minerals (ash) and low fat content, regardless of their anatomical location (LTL or HL muscles), which is consistent with the findings of other authors [38,39]. In the cited studies, the LD muscle of rabbits had higher total protein content, lower fat content, and similar ash content, compared with HL muscles. Hind leg muscles had higher IMF content, but it generally remained at a low level, which indicates that rabbit meat is lean [27].

### 4.2. Cholesterol, Vitamin A, and Vitamin E

In a study by Dalle Zotte [24], rabbit meat had the lowest cholesterol content among the most popular meat types. However, cholesterol concentration in rabbit meat may vary widely. According to Polak et al. [40], those differences result from the use of different analytical methods or samples from different carcass cuts as well as different interpretations of analytical data [41]. For instance, Gašperlin et al. [42] found that average cholesterol content was 76.6 mg/100 g of meat (*Longissimus lumborum* muscle + muscles of the abdominal wall + HL muscles), whereas Dalle Zotte et al. [43] noted average cholesterol content of 61.8 mg/100 g of meat (HL muscles). In a study by Loponte et al. [44], cholesterol concentration in the *Longissimus thoracis et lumborum* muscle of male CAL × NZW crosses raised in free-range areas and in open-air cages was 54.1 and 58.6 mg/100 g, respectively. The noted values were comparable with that determined in the present study in samples of the LTL muscle in male CAL rabbits (50.32 mg/100 g), but higher than that determined in females (43.43 mg/100 g). In this study, the genotype and sex of rabbits had no effect on the cholesterol content of meat, which is consistent with the findings of Gašperlin et al. [42] and Dalle Zotte et al. [43]. Polak et al. [40] demonstrated that cholesterol concentration was higher in the meat of male rabbits.

In the present experiment, the average content of vitamin A and vitamin E was 1.87 and 1.67 µg/g of meat, respectively. Vitamin A content was considerably higher in the muscles of females. Scant literature data are available on the levels of vitamins A and E in rabbit meat. Wognin et al. [45] demonstrated that the content of vitamin A and vitamin E in the HL muscles of rabbits ranged from 0.01 to 0.31 µg/100 g, and from 146.81 to 280.67 µg/100 g, respectively, depending on their diet. In a study by Dal Bosco et al. [46], the vitamin E content of rabbit meat was 2.15 mg/100 g.

### 4.3. Fatty Acid Profile

Unlike in polygastric animals, in monogastric animals (including rabbits), dietary fatty acids are incorporated into adipose tissue in practically unchanged form. Long-chain and medium-chain fatty acids, released during digestion, are catabolized and become a source of energy in adipocytes, whereas long-chain fatty acids are deposited as triglycerides [47]. Therefore, the fatty acid profile of rabbit meat may be directly related to the fatty acid profile of the animals’ diet [48], leading to differences in research findings. It should also be stressed that the fatty acid profile of rabbit fat differs due to its anatomical location [49].

In the present study, UFAs were the predominant fatty acid group in IMF. This is an important consideration because SFAs are associated with an increased risk of cardiovascular disease and their intake should be reduced [50]. Previous research [51] shows that the fatty acid profile of rabbit meat is determined by various factors, including genotype and sex which can exert various effects. In the current experiment, the meat of CAL rabbits was characterized by a more desirable fatty acid composition (higher concentrations of MUFAs and UFAs). In a study by Chodová et al. [52], the *Biceps femoris* muscle of rabbits had lower MUFA content and higher PUFA content in small breeds than in medium-sized and large breeds. Gašperlin et al. [42] demonstrated that neither genotype nor sex exerted significant effects on the fatty acid composition of rabbit meat. Dalle Zotte et al. [43] found that the HL muscles of crossbred rabbits originating from Vienna Blue and Burgundy sires did not differ significantly in their fatty acid profiles, except that the meat of rabbits derived from the Vienna Blue breed had a higher (*p* < 0.05) content of C10:0 fatty acid. However, the fatty acid composition of meat was affected by sex. The meat of female rabbits had a higher (*p* < 0.05) content of C18:3 n-3 fatty acid, a lower (*p* < 0.05) content of C16:0, C20:3 n-6 fatty acids, PUFAs and SFAs, and a lower (*p* < 0.05) SFA/UFA ratio. According to De Smet et al. [53], the effect of sex on the fatty acid profile of meat in cattle may be related to the possible influence of sex hormones on enzyme systems such as ∆9-desaturase. Such a relationship could also exist in rabbits, but further research is needed to validate this hypothesis.

### 4.4. Physicochemical Properties

The pH of meat has a considerable influence on many other meat quality attributes. According to Hulot and Ouhayoun [54], among biological factors (muscle, age, genotype, family), muscle type exerts the greatest effect on the differences in acidification. The differences in ultimate pH values between muscles can reach 0.7 units, and are due to differences in fiber typology. Ouhayoun et al. [55] demonstrated that in samples of 11 muscles, the ultimate pH values measured 22 h post mortem ranged from 5.71 to 6.00. According to Hulot and Ouhayoun [54], muscles from the carcass forequarters have higher ultimate pH values than those from the hindquarters. In turn, the latter have a higher pH than those from the loin. This is consistent with the results of the present study, which revealed that the average pH value was lower in the LTL muscle than in HL muscles.

The results of studies investigating the effect of sex on meat pH in rabbits are inconclusive. Yalçın et al. [56] and North et al. [36] found that meat pH was higher in males. However, Carrilho et al. [57], Dalle Zotte et al. [43], and Frunza et al. [28] demonstrated that sex had no effect on meat pH. Rabbit breed affected meat pH in a study by Wang et al. [58], whereas no such effect was observed by Dalle Zotte et al. [43]. The differences in meat acidity between males and females and rabbits of different genotypes, noted in the current experiment, could result from differences in muscle fiber composition or the glycolytic potential of muscle tissue. The proportion of aerobic fibers with lower glycogen content is higher in males, which affects meat acidity [59,60]. Regardless of the potential effects exerted by sex and genotype, the pH of rabbit meat can be influenced by various preslaughter factors [61].

The differences in the pH values of the muscles analyzed in this study affected the WHC of samples. The higher average pH values of meat from CAL rabbits and hind legs indicate that proteins far from their isoelectric points could bind more water, resulting in an increased WHC (lower drip loss determined by the filter paper press method).

The color of rabbit meat, similarly to other meat types, may be indirectly influenced by environmental factors associated with the housing and management system [62], preslaughter stress [63], and muscle activity [64]. According to Pascual and Pla [25], selection for growth rate also induces changes in the color of rabbit meat. The differences in the average values of color parameters L*, a*, and b*, reported in the literature, could be due to the fact that researchers used different instruments to measure meat color, and employed different methods to record reflectance measurements and calculate the above parameters [64].

In the present study, rabbit breed or genotype had no influence on meat color, which corroborates the findings of Dalle Zotte et al. [43] and North et al. [36]. The color parameters of meat (*Biceps femoris*) were affected by rabbit breed in experiments conducted by Chodová et al. [52] and Wang et al. [58]. Blasco et al. [65] reported differences in the values of L*, a*, and b* among rabbit breeds of different sizes, which however did not show a clear pattern.

Lazzaroni et al. [66] and Dalle Zotte et al. [43] noted a significant effect of sex on the values of color parameter b* in the *Longissimus lumborum* muscle, which were higher in female rabbits. According to Dalle Zotte et al. [43], the above resulted from a higher proportion of red slow-twitch fibers in the muscle of females, and lower activity of the enzyme lactate dehydrogenase.

In the current experiment, HL muscles were darker in color than the LTL muscle, most probably due to their higher pH. Moreover, HL muscles show higher activity than the *Longissimus* muscle, which contributes to higher myoglobin concentration and a darker red color.

### 4.5. Sensory Properties

According to Dalle Zotte [24], rabbit meat has a specific flavor profile, similar to that of wild game meat, which may not be well accepted by all consumers. Therefore, a slightly less intense aroma of meat from CAL rabbits, noted in this study, can be regarded as desirable. However, it should be stressed that an analysis of interaction effects revealed lower (*p* ≤ 0.05) aroma intensity only in the meat of males. Lower scores for aroma intensity could contribute to higher scores for aroma desirability in CAL rabbits.

In comparison with other types of meat, the juiciness of rabbit meat is assessed as medium or low [67], which was confirmed in the current study. The relatively low juiciness of meat from rabbits of both analyzed breeds was related to low IMF content. Intramuscular fat contributes to perceived juiciness by loosening the structure of connective tissue, which facilitates the release of juice during chewing and stimulates the salivary glands to produce saliva.

Meat tenderness is another sensory attribute related to IMF content [68]. However, the IMF content rabbit meat analyzed in this study was low, and its effect on tenderness could be disregarded. The observed differences in the tenderness and shear force values of LTL muscle samples cannot be attributed to the differences in meat acidity, which affects the activity of endogenous proteolytic enzymes of the calpain system [69]. In the present study, the pH values of LTL muscles were similar in FG and CAL rabbits (as confirmed by the absence of an interaction between the breed and muscle type). Therefore, it appears that meat tenderness could be affected by interbreed differences in muscle histology (the type and size of muscle fibers), which were observed by Chodová et al. [52]. However, Kozioł et al. [70], who investigated the effect of rabbit breed (Flemish Giant Gray, Californian Black, NZW, Popielno White, and Blanc de Termonde) on the shear force values and texture parameters of the *Longissimus lumborum* muscle, found that breed had a significant effect only on hardness, which was highest in Blanc de Termonde and lowest in Flemish Giant Gray rabbits. Ortiz Hernández and Rubio Lozano [37], who compared meat quality in four rabbit breeds (New Zealand, Rex, Chinchilla, and CAL), noted a significant difference in tenderness only between Rex and CAL breeds. According to Martínez-Álvaro and Hernández [71], an analysis of the quality of the *Longissimus dorsi* muscle should account for variation in the sensory attributes of samples collected along this muscle. In the cited study, considerable variation was noted particularly in the texture properties of rabbit meat (toughness, juiciness, and fibrousness), which reached approximately 10% between the caudal and cranial extremes. Variation was also found in the intensity of liver and rabbit odor and flavor along the LD muscle.

In the present study, sex had no effect on the analyzed sensory properties of rabbit meat. Differences in the sensory quality of meat from males and females were not reported by Frunza et al. [28], Gašperlin et al. [42], and Polak et al. [40]. Kozioł et al. [70], Ortiz Hernández and Rubio Lozano [37], and Trocino et al. [72] demonstrated that sex had no influence on the texture parameters of rabbit meat.

## 5. Conclusions

When CAL and FG rabbits are slaughtered at 91 days of age, differences can be expected in the proximate chemical composition, fatty acid profile, and sensory quality of their meat. The results of this study indicate that the meat of CAL rabbits was more mature at slaughter and had a more desirable fatty acid profile. Considerable differences were found in the chemical composition and physicochemical properties of the LTL muscle and HL muscles. Sex had no significant effect on the majority of the analyzed meat quality attributes. However, further research is needed to investigate why the meat of female rabbits had considerably higher vitamin A content.

## Figures and Tables

**Table 1:** Ingredients, chemical composition (% of dry matter), and energy value of diets.

Diet Ingredients	%
Wheat bran	36
Dehydrated alfalfa meal	25
Dried beet pulp	10
Sunflower husk	6
Rapeseed meal	5
Soybean meal	4
Barley	4
Palm cake	3
Rye bran	3
Molasses	1
Vitamin-mineral premix	1
Lime	1
Dicalcium phosphate	0.5
Salt (NaCl)	0.5
**Chemical composition of diets**	**% of dry matter**
Dry matter (%)	87.51
Total protein	16.48
Neutral detergent fiber (NDF)	23.28
N-free extract (NFE)	44.38
Crude fat	3.22
Lysine	0.79
Methionine and cystine	0.61
Tryptophan	0.18
Threonine	0.63
Calcium	0.79
Total phosphorus	0.44
Sodium	0.16
Metabolizable energy (MJ/kg)	14.50

**Table 2:** Chemical composition of meat (*Longissimus thoracis et lumborum*—LTL, hind leg—HL) from female and male Californian and Flemish Giant Gray rabbits (arithmetic means ± SEM).

Item	Breed		Sex		Muscle Type		SEM	*p*-Value		
	FG	CAL	Female	Male	LTL	HL		Breed	Sex	Muscle Type
Dry matter (%)	23.81 ^a^	25.30 ^b^	24.52	24.59	24.98 ^a^	24.13 ^b^	0.18	<0.001	0.784	0.003
Total protein (%)	21.96 ^a^	23.16 ^b^	22.58	22.54	23.33 ^a^	21.79 ^b^	0.17	<0.001	0.821	<0.001
Fat (%)	0.66	0.84	0.72	0.77	0.23 ^a^	1.26 ^b^	0.10	0.124	0.698	<0.001
Ash (%)	1.21	1.22	1.21	1.22	1.22	1.21	<0.01	0.055	0.389	0.180
Water/protein ratio (W/P)	3.48 ^a^	3.23 ^b^	3.35	3.35	3.22 ^a^	3.49 ^b^	0.03	<0.001	0.984	<0.001
Energy value (kJ)	393 ^a^	420 ^b^	406	407	400 ^a^	413 ^b^	3.67	<0.001	0.856	0.033
Cholesterol (mg/100 g)	44.54	49.21	43.43	50.32	x	x	2.30	0.277	0.117	x
Vitamin A (µg/g)	2.10	1.63	2.48 ^a^	1.25 ^b^	x	x	0.29	0.406	0.041	x
Vitamin E (µg/g)	1.59	1.75	1.76	1.58	x	x	0.08	0.382	0.320	x

FG—Flemish Giant Gray rabbits; CAL—Californian rabbits. SEM—standard error of the mean; x—not determined. Values in rows followed by different superscript letters, within experimental factors, are significantly different: ^a,b^—*p* ≤ 0.05.

**Table 3:** Percentages of saturated fatty acids in the total fatty acid pool in intramuscular fat (*Longissimus thoracis et lumborum*) in female and male Californian and Flemish Giant Gray rabbits (arithmetic means ± SEM).

Item	Breed		Sex		SEM	*p*-Value	
	FG	CAL	Female	Male		Breed	Sex
C10:0	0.18 ^a^	0.37 ^b^	0.21 ^a^	0.35 ^b^	0.04	0.001	0.020
C12:0	0.33 ^a^	0.47 ^b^	0.36	0.44	0.03	0.019	0.218
C14:0	2.93	3.01	2.95	3.00	0.04	0.402	0.589
C15:0	0.86 ^a^	0.73 ^b^	0.78	0.80	0.02	<0.001	0.347
C16:0	31.76	30.78	31.39	31.07	0.31	0.128	0.707
C17:0	1.13 ^a^	0.89 ^b^	1.02	0.98	0.04	<0.001	0.513
C18:0	11.42 ^a^	9.09 ^b^	10.74	9.59	0.41	0.002	0.116
C20:0	0.31 ^a^	0.24 ^b^	0.28	0.26	0.01	0.010	0.596
C22:0	0.47	0.29	0.39	0.35	0.05	0.061	0.855
SFAs	49.39 ^a^	45.85 ^b^	48.13	46.85	0.61	0.002	0.277

FG—Flemish Giant Gray rabbits; CAL—Californian rabbits. SEM—standard error of the mean; SFAs—saturated fatty acids. Values in rows followed by different superscript letters, within experimental factors, are significantly different: ^a,b^—*p* ≤ 0.05.

**Table 4:** Percentages of unsaturated fatty acids in the total fatty acid pool in intramuscular fat (*Longissimus thoracis et lumborum*) in female and male Californian and Flemish Giant Gray rabbits (arithmetic means ± SEM).

Item	Breed		Sex		SEM	*p*-Value	
	FG	CAL	Female	Male		Breed	Sex
C14:1	0.18 ^a^	0.15 ^b^	0.16	0.17	0.08	0.024	0.450
C16:1	0.96 ^a^	1.63 ^b^	1.36	1.27	0.11	0.001	0.466
C17:1	0.48	0.60	0.66	0.41	0.09	0.457	0.155
C18:1	22.77	24.21	23.68	23.36	0.43	0.094	0.600
C18:2 n-6	21.56	23.29	21.64	23.39	0.54	0.129	0.123
C18:3 n-3	2.12	2.29	2.22	2.21	0.10	0.479	0.976
C20:1	0.33	0.32	0.30 ^a^	0.35 ^b^	0.01	0.342	< 0.001
C20:2 n-6	0.23	0.19	0.21	0.21	0.02	0.326	0.859
C20:4 n-6	1.91 ^a^	1.42 ^b^	1.57	1.74	0.11	0.019	0.307
C20:5 n-3	0.06	0.05	0.07	0.04	0.01	0.765	0.226
MUFAs	24.73 ^a^	26.91 ^b^	26.16	25.56	0.51	0.027	0.418
PUFAs	25.88	27.25	25.71	27.59	0.61	0.324	0.149
UFAs	50.61 ^a^	54.15 ^b^	51.87	53.15	0.61	0.002	0.274

FG—Flemish Giant Gray rabbits; CAL—Californian rabbits. SEM—standard error of the mean; MUFAs—monounsaturated fatty acids; PUFAs—polyunsaturated fatty acids; UFAs—unsaturated fatty acids (UFAs = MUFAs + PUFAs). Values in rows followed by different superscript letters, within experimental factors, are significantly different: ^a,b^—*p* ≤ 0.05.

**Table 5:** Nutritional value of intramuscular fat (*Longissimus thoracis et lumborum*) in female and male Californian and Flemish Giant Gray rabbits (arithmetic means ± SEM).

Item	Breed		Sex		SEM	*p*-Value	
	FG	CAL	Female	Male		Breed	Sex
MUFA/SFA ratio	0.50 ^a^	0.59 ^b^	0.55	0.55	0.01	0.002	0.909
UFA/SFA ratio	1.03 ^a^	1.18 ^b^	1.08	1.14	0.03	0.002	0.247
PUFA/SFA ratio	0.53	0.60	0.54	0.59	0.02	0.063	0.139
DFA/OFA ratio	1.64	1.72	1.68	1.69	0.02	0.112	0.899
EFAs	23.68	25.59	23.86	25.60	0.60	0.139	0.169
Nutritional value *	1.08	1.08	1.10	1.06	0.02	0.914	0.440

FG—Flemish Giant Gray rabbits; CAL—Californian rabbits. SEM—standard error of the mean; UFAs—unsaturated fatty acids (MUFAs + PUFAs); SFAs—saturated fatty acids; MUFAs—monounsaturated fatty acids; PUFAs—polyunsaturated fatty acids; DFAs—hypocholesterolemic fatty acids (UFAs + C18:0); OFAs—hypercholesterolemic fatty acids (SFAs—C18:0); EFAs—essential fatty acids (C18:2 + C18:3); * (C18:0 + C18:1)/C16:0). Values in rows followed by different superscript letters, within experimental factors, are significantly different: ^a,b^—*p* ≤ 0.05.

**Table 6:** Physicochemical properties of meat (*Longissimus thoracis et lumborum—*LTL, hind leg—HL) from female and male Californian and Flemish Giant Gray rabbits (arithmetic means ± SEM).

Item	Breed		Sex		Muscle Type		SEM	*p*-Value		
	FG	CAL	Female	Male	LTL	HL		Breed	Sex	Muscle Type
pH	5.92 ^a^	6.14 ^b^	6.03	6.03	5.88 ^a^	6.18 ^b^	0.04	<0.001	0.817	<0.001
WHC (cm^2^)	6.41 ^a^	4.82 ^b^	5.44	5.79	6.08 ^a^	5.15 ^b^	0.22	<0.001	0.334	0.015
L*	62.56	61.32	61.54	62.34	62.80 ^a^	61.08 ^b^	0.35	0.059	0.218	0.011
a*	5.78	5.62	5.74	5.66	5.69	5.71	0.22	0.732	0.867	0.956
b*	15.36	15.20	15.19	15.36	15.71 ^a^	14.85 ^b^	0.12	0.415	0.382	<0.001
c*	16.48	16.22	16.29	16.41	16.75 ^a^	15.95 ^b^	0.16	0.382	0.655	0.010

FG—Flemish Giant Gray rabbits; CAL—Californian rabbits. SEM—standard error of the mean. WHC—water-holding capacity. Values in rows followed by different superscript letters, within experimental factors, are significantly different: ^a,b^—*p* ≤ 0.05.

**Table 7:** Sensory properties and shear force values of meat (*Longissimus thoracis et lumborum*) from female and male Californian and Flemish Giant Gray rabbits (arithmetic means ± SEM).

Item	Breed		Sex		SEM	*p*-Value	
	FG	CAL	Female	Male		Breed	Sex
Aroma-intensity	3.55	3.55	3.45	3.65	0.13	1.000	0.468
Aroma-desirability	4.90	4.75	4.85	4.80	0.08	0.319	0.736
Taste-intensity	4.20 ^a^	3.85 ^b^	3.95	4.10	0.10	0.042	0.357
Taste-desirability	4.70 ^a^	5.00 ^b^	4.85	4.85	0.06	0.021	1.000
Juiciness	3.00	3.10	3.05	3.05	0.03	0.176	1.000
Tenderness	4.60 ^a^	4.95 ^b^	4.80	4.75	0.08	0.046	0.762
Shear force value (N)	22.54 ^a^	14.72 ^b^	17.35	19.92	1.27	0.000	0.182

FG—Flemish Giant Gray rabbits; CAL—Californian rabbits. The five-point scale (5 points—most desirable, 1 point—least desirable) was used to evaluate each sensory trait. SEM—standard error of the mean. Values in rows followed by different superscript letters, within experimental factors, are significantly different: ^a,b^—*p* ≤ 0.05.

## References

[1] Trocino A., Cotozzolo E., Zomeño C., Petracci M., Xiccato G., Castellini C. (2019). Rabbit production and science: The world and Italian scenarios from 1998 to 2018. Ital. J. Anim. Sci..

[2] Animal Charity Evaluators. https://animalcharityevaluators.org/research/other-topics/trends-in-meat-production/#fao-tons-of-meat.

[3] Szendrö K., Szabó-Szentgróti E., Szigeti O. (2020). Consumers’ Attitude to Consumption of Rabbit Meat in Eight Countries Depending on the Production Method and Its Purchase Form. Foods.

[4] European Commission (2017). Directorate-General for Health and Food Safety.

[5] Dalle Zotte A. (2004). Avantage diététiques. Le lapin doit apprivoiser le consommateur. Viandes Prod. Carnes.

[6] Dalle Zotte A. (2014). Rabbit farming for meat purposes. Anim. Front..

[7] Dalle Zotte A., Szendrő Z. (2011). The role of rabbit meat as functional food: A review. Meat Sci..

[8] Hernández P., Dalle Zotte A., De Blas C., Wiseman J. (2010). Influence of diet on rabbit meat quality. Nutrition of the Rabbit.

[9] Parigi Bini R., Xiccato G., Cinetto M., Dalle Zotte A., Converso R. (1992). Effetto dell’età e del peso di macellazione e del sesso sulla qualità della carcassa e della carne cunicola. 1. Rilievi di macellazione e qualità della carcassa. Zootec. Nutr. Anim..

[10] Szendrő Z., Szendrő K., Dalle Zotte A. (2012). Management of Reproduction on Small, Medium and Large Rabbit Farms: A Review. Asian-Aust. J. Anim. Sci..

[11] Hernández P., Ariño B., Grimal A., Blasco A. (2006). Comparison of carcass and meat characteristics of three rabbit lines selected for litter size or growth rate. Meat Sci..

[12] Dalle Zotte A., Paci G. (2014). Rabbit growth performance, carcass traits and hind leg bone characteristic as affected by sire genetic origin, slaughter season, parity order and gender in an organic production system. Anim. Sci. Pap. Rep..

[13] De Blas C., Mateos G.G., De Blas C., Wiseman J. (2010). Feed formulation. Nutrition of the Rabbit.

[14] Association of Official Analytical Chemists (AOAC) (1990). Official Methods of Analysis.

[15] Daszkiewicz T., Janiszewski P. (2020). The effect of sex on the quality of meat from farmed pheasants (*Phasianus colchicus*). Anim. Sci. J..

[16] Rhee K.S., Dutson T.R., Smith G.C., Hostetler R.L., Reiser R. (1982). Cholesterol Content of Raw and Cooked Beef Longissimus Muscles with Different Degrees of Marbling. J. Food Sci..

[17] Hőgberg A., Pickova J., Babol J., Andersson K., Dutta P.C. (2002). Muscle lipids, vitamins E and A, and lipid oxidation as affected by diet and RN genotype in female and castrated male Hampshire crossbreed pigs. Meat Sci..

[18] Xu Z. (2008). Comparison of extraction methods for quantifying vitamin E from animal tissues. Bioresour. Technol..

[19] Commission Internationale de l’Éclairage (CIE) (1978). Recommendations on Uniform Color Spaces-color Difference Equations.

[20] Van Oeckel M.J., Warnants N., Boucqueé C.V. (1999). Comparison of different methods for measuring water holding capacity and juiciness of pork versus on-line screening methods. Meat Sci..

[21] Żegarska Z., Jaworski J., Borejszo Z. (1991). Evaluation of the Peisker modified method for extracting methyl esters from fatty acids. Acta Acad. Agricult. Tech. Olst..

[22] Honikel K.O. (1998). Reference methods for the assessment of physical characteristics of meat. Meat Sci..

[23] Daszkiewicz T., Kubiak D., Winarski R., Koba-Kowalczyk M. (2012). The effect of gender on the quality of roe deer (*Capreolus capreolus* L.) meat. Small Rumin. Res..

[24] Dalle Zotte A. (2002). Perception of rabbit meat quality and major factors influencing the rabbit carcass and meat quality. Livest. Prod. Sci..

[25] Pascual M., Pla M. (2007). Changes in carcass composition and meat quality when selecting rabbits for growth rate. Meat Sci..

[26] Ariño B., Hernández P., Pla M., Blasco A. (2007). Comparison between rabbit lines for sensory meat quality. Meat Sci..

[27] Hernández P. Enhancement of nutritional quality and safety in rabbit meat. Proceedings of the 9th World Rabbit Congress.

[28] Frunza G., Simeanu D., Mircea Pop I., Boisteanu P.C., Stefan M. (2019). Contributions on the Sensorial, Physico-Chemical and Nutritional Characterization of Meat in Flemish Giant Rabbit Breed. Rev. Chim..

[29] Gondret F., Juin H., Mourot J., Bonneau M. (1998). Effect of Age at Slaughter on Chemical Traits and Sensory Quality of *Longissimus lumborum* Muscle in the Rabbit. Meat Sci..

[30] Pla M., Pascual M., Ariño B. (2004). Protein, fat and moisture content of retail cuts of rabbit meat evaluated with the NIRS methodology. World Rabbit Sci..

[31] Belichovska D., Belichovska K., Pejkovski Z., Uzunoska Z. (2017). Effect of genotype on physico-chemical characteristics of rabbit meat. Meat Technol..

[32] Maj D., Bieniek J., Łapa P. (2008). Meat quality of New Zealand White and Californian rabbits and their crosses. Med. Wet..

[33] Piórkowska M. (2008). Slaughter value of rabbits of different genotypes. Rocz. Inst. Przemysłu Mięsnego I Tłuszczowego.

[34] Martínez-Alvaro M., Hernández P., Agha S., Blasco A. (2018). Correlated responses to selection for intramuscular fat in several muscles in rabbits. Meat Sci..

[35] Baiomy A.A., Hassanien H.H.M. (2011). Effect of breed and sex on carcass characteristics and meat chemical composition of New Zealand White and Californian rabbits under upper egyptian environment. Egypt. Poult. Sci..

[36] North M.K., Dalle Zotte A., Hoffman L.C. (2018). The effects of quercetin supplementation on New Zealand White grower rabbit carcass and meat quality—A short communication. Meat Sci..

[37] Ortiz Hernández J.A., Rubio Lozano M.S. (2001). Effect of breed and sex carcass yield and meat quality. World Rabbit Sci..

[38] Mattioli S., Cardinali R., Balzano M., Pacetti D., Castellini C., Dal Bosco A., Frega N.G. (2017). Influence of dietary supplementation with prebiotic, oregano extract, and vitamin E on fatty acid profile and oxidative status of rabbit meat. J. Food Qual..

[39] Metzger S., Odermatt M., Szabó A., Radnai I., Biró-Németh E., Nagy I., Szendrö Z. (2011). Effect of age and body weight on carcass traits and meat composition of rabbits. Arch. Tierz..

[40] Polak T., Gašperlin L., Rajar A., Žlender B. (2006). Influence of genotype lines, age at slaughter and sexes on the composition of rabbit meat. Food Technol. Biotechnol..

[41] Arneth W., Alahmad H. (1995). Cholesterol—Its determination in muscle and adipose-tissue and in offal using HPLC. Fleischwirtschaft.

[42] Gašperlin L., Polak T., Rajar A., Skvarèa M., Zlender B. (2006). Effect of genotype, age at slaughter and sex on chemical composition and sensory profile of rabbit meat. World Rabbit Sci..

[43] Dalle Zotte A., Cullere M., Alberghini L., Catellani P., Paci G. (2016). Proximate composition, fatty acid profile, and heme iron and cholesterol content of rabbit meat as affected by sire breed, season, parity order, and gender in an organic production system. Czech J. Anim. Sci..

[44] Loponte R., Secci G., Mancini S., Bovera F., Panettieri V., Nizza A., Di Meo C., Piccolo G., Paris G. (2018). Effect of the housing system (free-range vs. open air cages) on growth performance, carcass and meat quality and antioxidant capacity of rabbits. Meat Sci..

[45] Wognin L.R.M.F., Otchoumou K.A., Yao K.F., Niamke S. (2018). Improving the nutritive value and sensory quality of rabbit meat by using leafy vegetables as feedstuffs. J. Anim. Plant Sci..

[46] Dal Bosco A., Castellini C., Bernardini M. (2001). Nutritional quality of rabbit meat as affected by cooking procedure and dietary vitamin E. J. Food Sci..

[47] Gondret F., Mourot J., Lebas F., Bonneau M. (1998). Effects of dietary fatty acids on lipogenesis and lipid traits in muscle, adipose tissue and liver of growing rabbits. Anim. Sci..

[48] Peiretti P.G., Mussa P.P., Prola L., Meineri G. (2007). Use of different levels of false flax (*Camelina sativa* L.) seed in diets for fattening rabbits. Livest. Sci..

[49] Yonkova P.Y., Mihaylova G.S., Ribarski S.S., Doichev V.D., Dimitrov R.S., Stefanov M.G. (2017). Fatty acid composition of subcutaneous and visceral fat depots in New Zealand White rabbits 2017. Bulg. J. Vet. Med..

[50] Briggs M.A., Petersen K.S., Kris-Etherton P.M. (2017). Saturated Fatty Acids and Cardiovascular Disease: Replacements for Saturated Fat to Reduce Cardiovascular Risk. Healthcare.

[51] Peiretti P.G. (2012). Effects of dietary fatty acids on lipid traits in the muscle and perirenal fat of growing rabbits fed mixed diets. Animals.

[52] Chodová D., Tůmová E., Martinec M., Bízková Z., Skřivanová V., Volek Z., Zita L. (2014). Effect of housing system and genotype on rabbit meat quality. Czech J. Anim. Sci..

[53] De Smet S., Raes K., Demeyer D. (2004). Meat fatty acid composition as affected by fatness and genetic factors: A review. Anim. Res..

[54] Hulot F., Ouhayoun J. (1999). Muscular pH and related traits in rabbits: A review. World Rabbit Sci..

[55] Ouhayoun J., Daudin J.D., Raynal H. (1990). Technologie de l’abattage du lapin. Influence de la température de l’air de réfrigération sur les pertes d’eau et sur l’acidification musculaire. Viandes Prod. Carnés.

[56] Yalçın S., Onbaşılar E.E., Onbaşılar İ. (2006). Effect of sex on carcass and meat characteristics of New Zealand White rabbits aged 11 weeks. Asian-Aust. J. Anim. Sci..

[57] Carrilho M.C., Campo M.M., Olleta J.L., Beltrán J.A., López M. (2009). Effect of diet, slaughter weight and sex on instrumental and sensory meat characteristics in rabbits. Meat Sci..

[58] Wang J., Su Y., Elzo M.A., Jia X., Chen S., Lai S. (2016). Comparison of carcass and meat quality traits among three rabbit breeds. Korean J. Food Sci. An..

[59] Dalle Zotte A., Ouhayoun J., Parigi Bini R., Xiccato G. (1996). Effect of age, diet and sex on muscle energy metabolism and on related physicochemical traits in the rabbit. Meat Sci..

[60] Lefaucheur L. (2010). A second look into fibre typing—Relation to meat quality. Meat Sci..

[61] Składanowska-Baryza J., Stanisz M. (2019). Pre-slaughter handling implications on rabbit carcass and meat quality–A review. Ann. Anim. Sci..

[62] Dal Bosco A., Castellini C., Mugnai C. (2002). Rearing rabbits on a wire net floor or straw litter: Behaviour, growth and meat qualitative traits. Livest. Prod. Sci..

[63] Maria G.A., Liste G., Villarroel M., Buil T., Sañudo C., Olleta J.L., Lόpez M. Effect of transport time and season on aspects of rabbit meat quality. Proceedings of the 50th International Congress of Meat Science and Technology.

[64] Dalle Zotte A., Princz Z., Metzger S., Szabό A., Radnai I., Biró-Németh E., Orova Z., Szendrő Z. (2009). Response of fattening rabbits reared under different housing conditions. 2. Carcass and meat quality. Livest. Sci..

[65] Blasco A., Nagy I., Hernández P. (2018). Genetics of growth, carcass and meat quality in rabbits. Meat Sci..

[66] Lazzaroni C., Biagini D., Lussiana C. (2009). Different rearing systems for fattening rabbits: Performance and carcass characteristics. Meat Sci..

[67] Rødbotten M., Kubberd E., Lea P., Ueland Ø. (2004). A sensory map of the meatuniverse. Sensory profile of meat from 15 species. Meat Sci..

[68] Hocquette J.F., Gondret F., Baéza E., Médale F., Jurie C., Pethick D.W. (2010). Intramuscular fat content in meat-producing animals: Development, genetic and nutritional control, and identification of putative markers. Animal.

[69] Bhat Z.F., Morton J.D., Mason S.L., Bekhit A.E.-D.A. (2018). Role of calpain system in meat tenderness: A review. Food Sci. Hum. Wellness.

[70] Kozioł K., Siudak Z., Pałka S., Kmiecik M., Otwinowska-Mindur A., Migdał Ł., Bieniek J. (2017). The effect of breed and sex on the texture of rabbit meat. Sci. Ann. Pol. Soc. Anim. Prod..

[71] Martínez-Álvaro M., Hernández P. (2018). Evaluation of the sensory attributes along rabbit loin by a trained panel. World Rabbit Sci..

[72] Trocino A., Xiccato G., Queaque P.I., Sartori A. (2003). Effect of transport duration and gender on rabbit carcass and meat quality. World Rabbit Sci..

